# ZCCHC12 promotes the progression of osteosarcoma via PI3K/AKT pathway

**DOI:** 10.18632/aging.204296

**Published:** 2022-09-19

**Authors:** Yong Cui, Yong-Yong Dong

**Affiliations:** 1 Department of Orthopedics, Jincheng People's Hospital, Jincheng 048026, Shanxi Province, China

**Keywords:** OS, ZCCHC12, PI3K, AKT

## Abstract

Researchers have reported that zinc finger CCHC domain containing 12 gene (ZCCHC12) plays a role in the progression and tumorigenesis of papillary thyroid cancer. However, the biological role of ZCCHC12 in osteosarcoma (OS) remains unknown. ZCCHC12 was highly upregulated in OS cell lines according to our present study. Also, our subsequent assays demonstrated that ZCCHC12 enhanced the proliferation, tumor growth and migration of OS cells. Moreover, the epithelial-mesenchymal transition (EMT) of OS cells was also promoted by ZCCHC12. In addition, downregulation of ZCCHC12 induced apoptosis and S-phase arrest in OS cells. Then, our study indicated that ZCCHC12 exerts its oncogenic function in OS cells by activating the PI3K/AKT pathway. Inhibition of the PI3K/AKT pathway greatly limits the oncogenic function of ZCCHC12 in OS cells. Also, overexpression of ZCCHC12 promotes tumor growth *in vivo*. Altogether, our study suggests ZCCHC12 promotes OS cells progression by activating the PI3K/AKT pathway. The ZCCHC12 gene may be a novel diagnostic and therapeutic target for OS.

## INTRODUCTION

Osteosarcoma (OS), one of the most frequent primary tumors, is characterized by direct production of bone or bone-like tissue by the cancerous cells [1, 2]. The osteosarcoma is locally invasive and can metastasize at an early stage. Typically, osteosarcoma occurs in adolescents in the metaphysis of long bones, such as the proximal tibia and distal femur. The five-year survival rate of osteosarcoma patients with lung metastases was only 19% [3–6]. Although the diagnosis and treatment of osteosarcoma has made progress compared to the past few decades [7–14], the prognosis for patients with metastatic or recurrent cancer is still poor [15]. So, it is pivotal to study the mechanism underlying the progression of osteosarcoma and develop novel therapy targets of osteosarcoma.

As a coactivator of transcription, Zinc finger CCHC domain containing 12 gene (ZCCHC12) contains a zinc finger domain [16, 17]. ZCCHC12 encodes a downstream effector of bone morphogenetic protein (BMP) pathway and activating BMP pathway through interaction with SMAD1 and association with CBP [18]. A variant in ZCCHC12 may play a role in X-linked cognitive disability [19]. Studies have found that ZCCHC12 is associated with the epithelial-mesenchymal transition (EMT) of tumor cells [20]. Despite this, no functional role has been revealed for ZCCHC12 in osteosarcoma occurrence or development. Thus, investigation of the influence of ZCCHC12 on osteosarcoma tumorigenesis is crucial.

As part of our present study, we investigated the underlying biochemical mechanisms of ZCCHC12 and its impact on osteosarcoma. Analysis of whole transcriptome in osteosarcoma showed that osteosarcoma exhibits an upregulation of ZCCHC12 in comparison to normal bone tissues. Meanwhile, we found that osteosarcoma cells may be induced to proliferate and migrate by ZCCHC12. Cell cycle arrest occurs when ZCCHC12 is knocked down in osteosarcoma cells. As a result of activating the PI3K/AKT pathway, ZCCHC12 promotes osteosarcoma tumorigenesis, while inhibition of PI3K/AKT pathway limits its effectiveness in osteosarcoma. Moreover, our *in vitro* research showed that ZCCHC12 to promote tumor growth. Our study indicates that ZCCHC12 may be a novel therapy target of osteosarcoma.

## MATERIALS AND METHODS

### Bioinformatics analysis

The sequencing data obtained from the GEO database (GSE99671) includes 18 pairs of normal bone tissue samples and bone tumor samples. On the sequencing data, a difference analysis was performed by R using the limma package.

### Cell lines and cell culture

HOS, 143B, Saos-2, U2OS, Hfob1.19 and MG63 were purchased from Chinese Academy of Sciences Cell Bank (Shanghai, China). A Dulbecco’s modified eagle medium (DMEM, Thermo Scientific, USA) with 10% fetal bovine serum (FBS, Thermo Fisher, USA), and 100 U/ml of penicillin and 100 *g/mL of streptomycin (Invitrogen) was used for cell culturing under 5% CO2 at 37°C. Cells were passaged when the density reaches 90%.

### CCK-8 assay

The cell proliferation status was evaluated with the Cell Counting Kit-8 (CCK-8, Dojindo Laboratories, Japan) assay. Transfected HOS and 143B cells were plated at 2000 cells per well in 96-well plates. Respectively, the cell state was detected with CCK8 reagents 24, 48, 72, 96 hours after seeding. After incubating with CCK-8 reagents in dark at 37°C for 2 hours, SpectraMax 190 Microplate Reader was used to measure the optical density at 450 nm.

### Apoptosis assay

Following three-time washing with PBS, the transfected HOS cells were collected. Then we added 100 μL 1× binding buffer containing 5 μL of annexin V-FITC (Beyotime, China) and 10 μL of propidium iodide (PI) to resuspend cells. Incubated at room temperature without light for 20 min with binding buffer, flow cytometry (Beckman Coulter Diagnostics CytoFlex, USA) was used to detect the apoptosis rate of HOS cells.

### Colony-forming assay

After transfection, 143B and HOS cells were grown for 9 days in six-well plates at a density of 500 cells per well. Cells were washed three times with PBS before being fixed for 20 minutes with 4% paraformaldehyde (Beyotime, China). The fixed cells were washed three times with PBS before being stained for 25 minutes with 1% crystal violet. After drying up, cells were photographed and colonies counted.

### Lentivirus transfection

Knockdown and overexpression lentiviruses were designed and constructed by Jiman Biotechnology (Shanghai) Co., Ltd. Lentivirus transfection was performed at a cell density of 25%. The original medium was discarded, and serum-free medium containing 5 ug/ml polybrene was added to the cells, followed by the lentivirus suspension. The medium was switched to normal cell culture medium 24 hours following transfection. Two days after virus transfection, cell selection was performed using puromycin. Cells in suspension were removed, where adherent cells were considered lentivirus transfected.

### si-RNA transfection

Plasmids were constructed by Jiman (Shanghai) Co., Ltd. HOS cells were cultured in six-well plates in advance, and si-RNA transfection was performed at 50% cell density. For each well of cells, following diluting si-RNA with 125 ul of serum-free medium, the mixture was let stand for 5 minutes. Dilute 5 ul of lipo 2000 reagent with 125 ul of serum-free medium, mix gently and let stand for 5 minutes. Following the mixing of the two mixtures, 15 minutes of standing was allowed at room temperature. PBS was used to wash the six-well plate once before replacing the medium with serum-free medium. Gently added the transfection reagent-plasmid mixture into the six-well plate and cultured for 6 hours. Following the removal of the medium, the normal medium was substituted.

### Cell cycle analyses

PBS was used to wash the cells three times after removing the cell culture medium. Then, trypsin was used for cell digesting. Medium containing FBS was added 3 min after digestion. Then we transfer the cells to a centrifuge tube and centrifuge at 1000 g for 3 min. After removing the supernatant, we resuspend the cells in PBS, and then centrifuge again. The pre-cooled 70% ethanol was added to each centrifuge tube after removing PBS. Centrifuge tubes were rotated overnight at 4°C. Then, ethanol was removed from fixated cells by centrifugation at 1000 g for five minutes. Each sample was prepared with propidium iodide staining solution according to the dosage of 0.5 ml staining buffer, 25 ul propidium iodide staining solution (20×) and 10 ul RNase A (50×). Flow cytometry (Beckman Coulter Diagnostics CytoFlex, USA) was used to determine cell cycle after 30 minutes of incubation at 37°C without light.

### qRT-PCR

After aspirating the culture medium, HOS and 143B cells were washed with 1 ml of PBS per well. Subsequently, Trizol (Invitrogen, USA) was added, the HOS and 143B cells were gently pipetted 3–5 times and lysed on ice for 5 minutes. Following scraping off the cells, lysates were transferred to a new centrifuge tube. Chloroform was added to each centrifuge tube after letting the lysis buffer stand at room temperature for 5 minutes. The trizol and chloroform mixture was left to stand for 3 minutes at room temperature after mixing thoroughly. Then, the centrifuge tubes were centrifuged at 12,000 g for 15 minutes. Removing the clear liquid from the upper layer and transferred it into a new centrifuge tube. The mixture was centrifuged for 10 minutes at 12000 g after adding isopropanol and letting it stand for 10 minutes. Pouring off the liquid in the tube and RNA precipitate can be seen at the bottom of the centrifuge tube. Next, 75% ethanol was added to wash the RNA precipitate. After centrifugation, 20 ul of DEPC water was added to each tube. The RNA solution was loaded to Nano Drop2000 to detect the concentration and A260/A280 ratio. PrimeScript RT reagent (TAKARA, RR036A) was used for reverse transcription of RNA. qRT-PCR was performed with Hieff^®^ qPCR SYBR Green Master Mix (Low Rox Plus) reagent from Yisheng Biotechnology (Shanghai) Co., Ltd following the manufacturer’s instructions.

### Transwell assays

2 × 10^5^ 143B or HOS cells were plated in the upper chamber of a 24-well transwell chamber. A serum-free medium was used to culture the cells for 24 hours at 37°C. Afterward, cells were washed twice with PBS and fixed for 20 minutes with 4% paraformaldehyde. At 37°C, the lower surface of the chamber was stained with 0.1% crystal violet for 30 minutes while the upper surface was removed gently using cotton swabs. We counted traversed cells under a microscope in 3 random fields.

### Wound healing assays

HOS and 143B cells were seeded into six-well plate. The cells were scratched with a 200 *l plastic pipette tip after reaching 90% density. Afterwards, the cells were washed with PBS and cultivated in normal medium. Images were taken at 0 h, 12 h and 24 h after scratch under a microscope at 3 random fields.

### Western blotting

HOS and 143B Cells were lysed with Radio immunoprecipitation assay buffer (RIPA, Beyotime) in presence of Phenylmethanesulfonyl fluoride (PMSF, Beyotime) on ice for half an hour. The 10% SDS-PAGE polyacrylamide gels were made and used for lysates separating. Proteins on the gels were then transferred to polyvinylidene fluoride (PVDF) membranes (0.22 μm, Beyotime) and blocked with proteins-free quick blocking buffer (Epizyme, PS108P) for 15 minutes at 37°C. Incubation was conducted overnight with primary antibodies on PVDF membranes at 4°C. Following three TBST washes, the membranes were incubated for 1 hour at 37°C with horseradish peroxidase (HRP)-conjugated secondary antibodies (Beyotime). ECL reagent (Epizyme) was used for the detection of antibody-antigen complexes.

### Subcutaneous xenografts

The Ethics Committee at our hospital approved our animal studies. Male nude mice (aged 4 weeks, weighting 18–20 g) were purchased from Jihui Laboratory Animal Breeding Co., Ltd. and housed in the SPF animal room of local hospital. Each cage housed five mice, and the animal room was placed under a 12-hour light/12-hour dark cycle. The temperature in the animal room was constant at 21°C. Each group contained five mice. Four independent groups were formed. The right axilla of each mouse was subcutaneously injected with 10^6^ 143B cells transfected with Vector, LV-ZCCHC12, Vector-shRNA or LV-shZCCHC12. By caliper, tumor volumes were measured on a weekly basis (0.5 × width^2^ × length). Four weeks after injection, mice were sacrificed, and tumors were dissected and weighed.

### Immunohistochemistry

Tumor tissues were embedded in paraffin after fixation with 4% paraformaldehyde. After deparaffinizing tissue slides in xylene, they were rehydrated in alcohol. 3% hydrogen peroxide was used for endogenous peroxidase blocking for IHC. Then 0.1 M citric sodium buffer was used under microwave for antigen retrieval. Tumor sections were blocked with 5% BSA for an hour and then incubated overnight at 4°C with the primary antibody. After washing three times with PBS, the antibody binding was detected with HRP-DAB kit (Maxvision^TM2^ HRP-Polymer anti-Rabbit IHC Kit) and the nucleus was counterstained with haematoxylin. Olympus microscopes were used to acquire the images.

### Statistical analysis

Data is expressed as mean ± S.D. The Gaussian distribution of the data is tested by Shapiro-Wilk criterion. A two-tailed Student’s *t* test was used to determine whether a continuous variable differed statistically significantly between two groups. The Tukey’s test was applied after the one-way analysis of variance for comparisons between three or more groups. Statistical significance was determined by *P* < 0.05.

## RESULTS

### ZCCHC12 was upregulated in human OS tissues and cell lines

**Figure 1:**
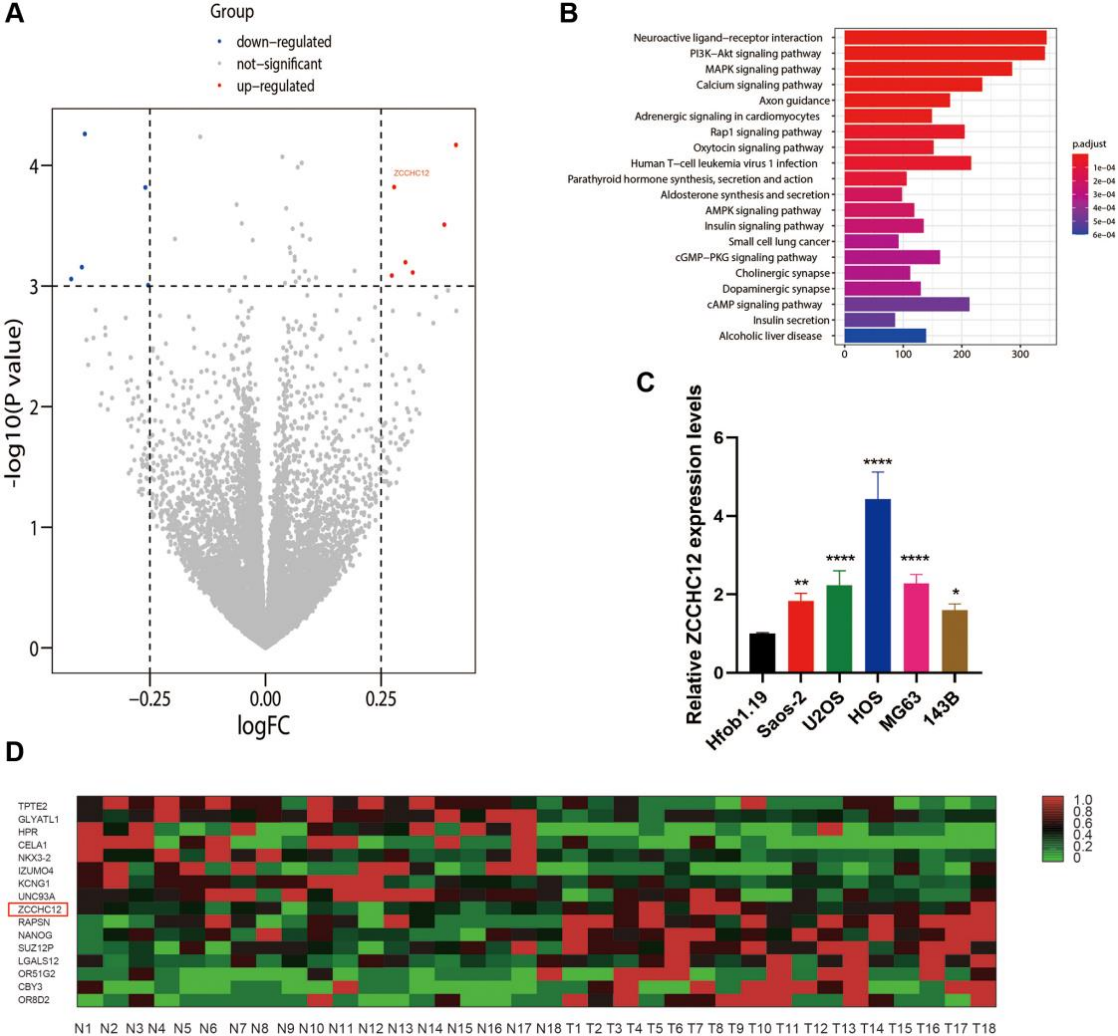
**ZCCHC12 was upregulated in OS.** (**A**) Differences in gene expression between OS and normal bone tissues represented by a volcano plot. (**B**) KEGG enrichment analysis indicated that PI3K/AKT signaling pathways had been significantly altered. (**C**) ZCCHC12 expression was detected in OS cell lines by qRT-PCR. (**D**) Heatmap of differential gene expression in OS and normal bone tissues. *n* = 5; ^*^P < 0.05, ^**^P < 0.01, ^***^P < 0.001, ^****^P < 0.0001.

ZCCHC12 was identified by analyzing a previously performed RNA-Sequencing analysis (GEO, ID: GSE99671) in 18 OS tissues and matched adjacent non-tumor tissues. The expression of ZCCHC12 was significantly increased in OS cells and tissues compared with non-cancer cells and tissues (Figure 1A, 1C and 1D). The top 20 significantly enriched signaling pathways from KEGG enrichment analysis were shown in Figure 1B, among which there were several well-known cancer-related pathways, such as PI3K/AKT signaling pathway, MAPK signaling pathway, et al.

### ZCCHC12 promoted OS cell proliferation and migration

**Figure 2:**
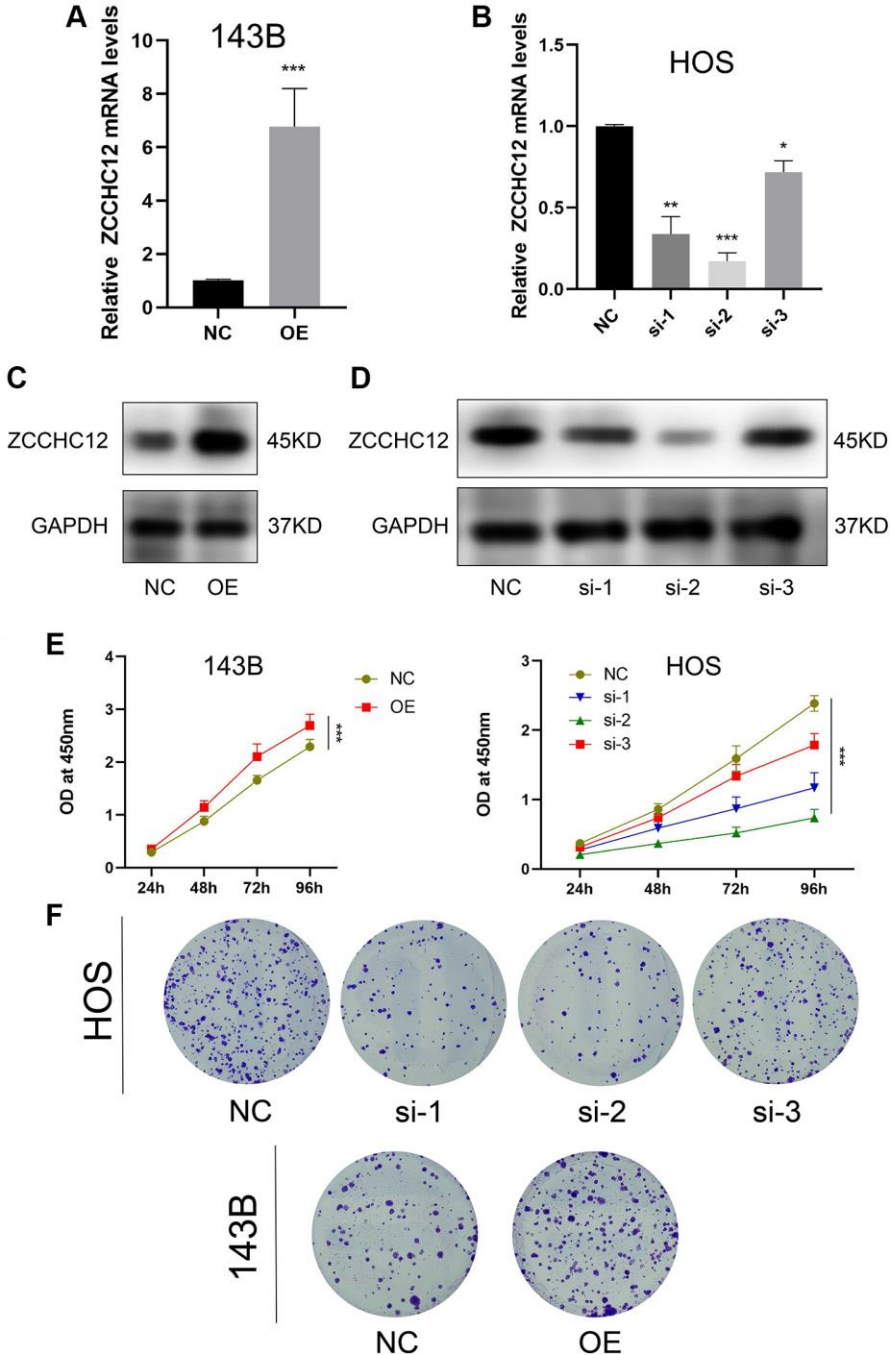
**ZCCHC12 promoted OS cell proliferation.** (**A** and **B**), qRT-PCR was applied to verify the overexpression and knockdown efficiency of ZCCHC12. (**C** and **D**), western blot analysis was performed to examine the expression of ZCCH12 in OS cells. (**E**) CCK8 analyses of the OS cell proliferation with ZCCHC12 overexpression or knockdown. (**F**) Colony formation assay analysis of cell proliferation after ZCCHC12 knockdown or overexpression in OS cells. *n* = 5; ^*^P < 0.05, ^**^P < 0.01, ^***^P < 0.001.

**Figure 3:**
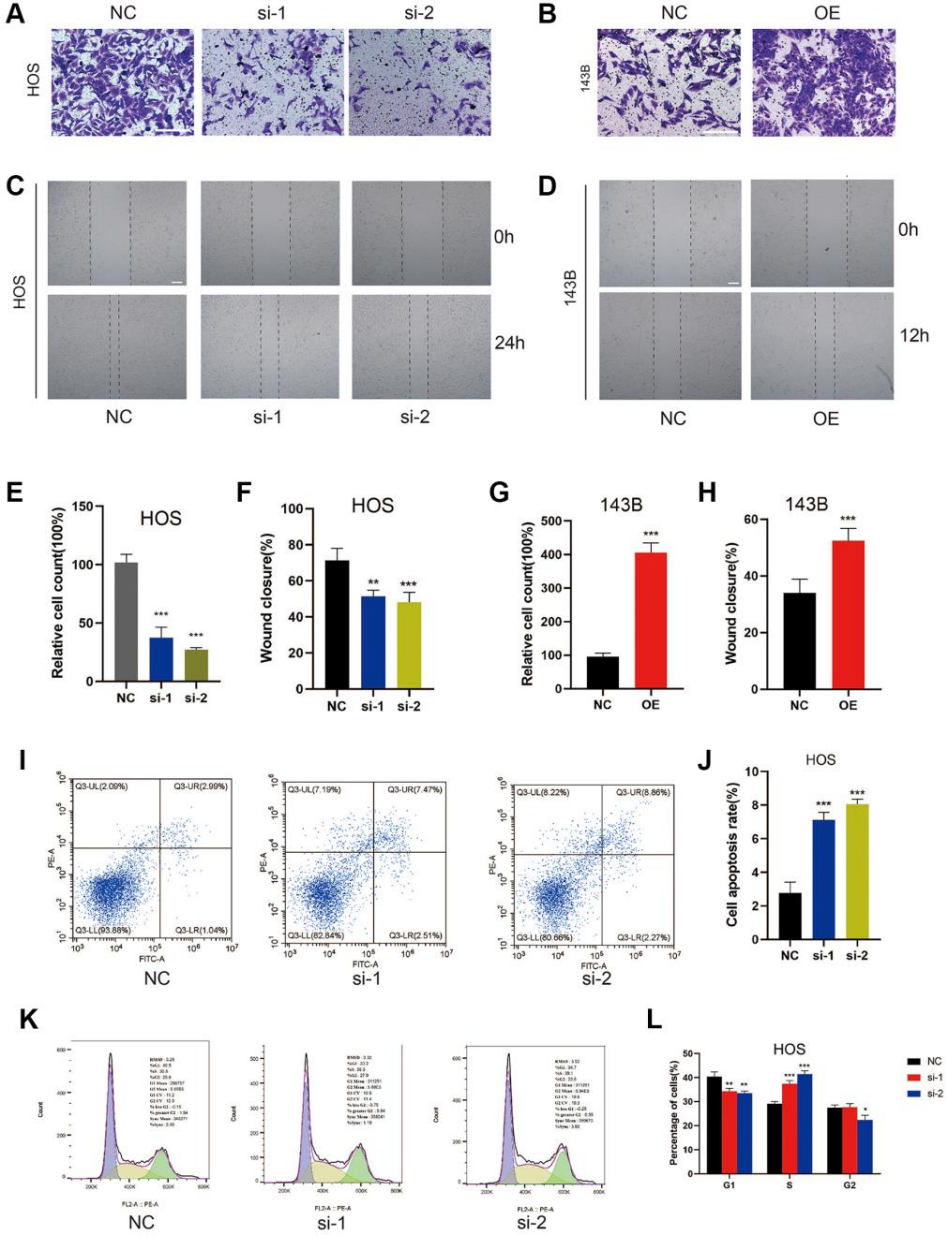
**ZCCHC12 facilitated cell migration and inhibition of it induced cell apoptosis and S-phase arrest in OS cells.** (**A**–**D**), migration ability of cells was detected by Transwell migration assays (**A** and **B**) and wound-healing assays (**C** and **D**). (**E**–**H**), the quantifications of cell migration were presented by the column chart. (**I**) Flow cytometry images of the cell apoptosis and (**J**) column bar graph of apoptotic cells. (**K**) Cell cycle in HOS cells with ZCCHC12 knockdown and (**L**) percentages of cells in each cell cycle phase are shown in the bar graph. scale bar: 200 μm. *n* = 5; ^*^P < 0.05, ^**^P < 0.01, ^***^P < 0.001.

We performed gain-of-function and loss-of-function assays to determine the biological role of ZCCHC12 in OS. The highest-ZCCHC12-expressing cell line, HOS, was selected to construct ZCCHC12-knockdown cell model by transfection with specific siRNAs; meanwhile, the lowest-ZCCHC12-expressing cell line, 143B, was used to establish ZCCHC12-overexpression cell model by transfection with full-length ZCCHC12 lentivirus. Western blots and qRT-PCR were used to validate knockdown and overexpression efficiency (Figure 2A–2D). As shown in Figure 2E and 2F, ZCCHC12 knockdown significantly reduced OS cell growth and colony formation, whereas ZCCHC12 overexpression had the opposite effect. Moreover, we found that ZCCHC12 enhanced the migration of OS cells. Transwell migration assays showed that ZCCHC12 knockdown dramatically inhibited OS cells’ mobility (Figure 3A and 3E), whereas ZCCHC12 overexpression played the opposite role (Figure 3B and 3G). Similar to Transwell assays, wound healing assays also showed similar results (Figure 3C, 3D, 3F and 3H). Based on these findings, ZCCHC12 enhanced OS cells proliferation and migration *in vitro*.

### ZCCHC12 knockdown induced OS cell apoptosis and cell cycle arrest in S-phase

By using flow cytometry, we assessed ZCCHC12 knockdown’s impact on OS cell apoptosis and cell cycle regulation. As shown in Figure 3I and 3J, the suppression of ZCCHC12 significantly increased the level of cell apoptosis. Meanwhile, ZCCHC12 knockdown led to an increased S-phase cell fraction, indicating a pronounced cell cycle arrest in S-phase (Figure 3K and 3L). Considering the results above, we assumed that ZCCHC12 regulated OS cell proliferation through regulation of apoptosis and cell cycle.

### ZCCHC12 accelerated EMT in OS cells

**Figure 4:**
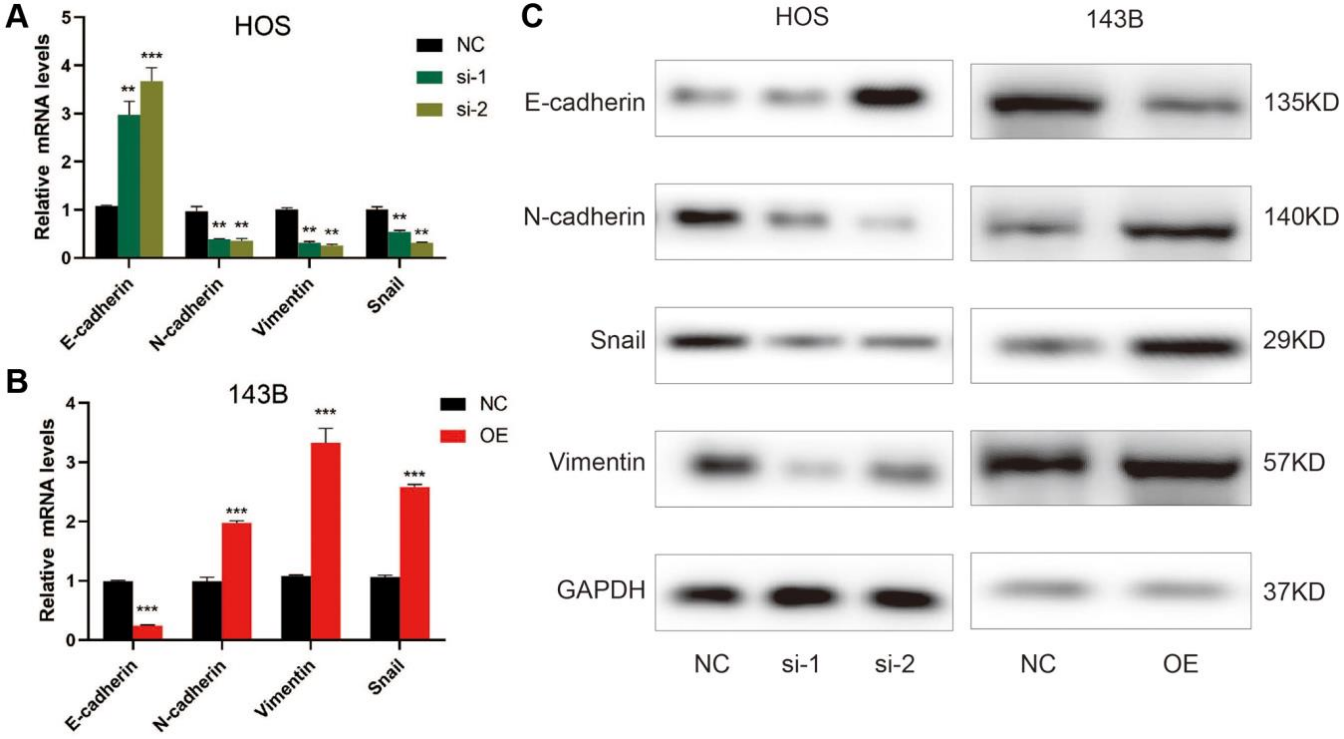
**ZCCHC12 promoted OS cell EMT progression.** (**A**–**C**), qRT-PCR (**A** and **B**) and western blot analysis (**C**) were performed to examine EMT-related markers in OS cells after ZCCHC12 knockdown or overexpression. *n* = 5; ^**^P < 0.01, ^***^P < 0.001.

Given that EMT is closely associated with the motility of cancer cells [21], we examined ZCCHC12’s effect on EMT-related markers by qRT-PCR and western blot. As shown in Figure 4A–4C, E-cadherin was upregulated after ZCCHC12 knockdown, while N-cadherin, Snail and Vimentin were downregulated. Moreover, ZCCHC12 overexpression had the opposite effect. The findings indicate that ZCCHC12 plays a critical role in the EMT of OS.

### ZCCHC12 facilitated OS progression via activating PI3K/AKT signaling pathway

**Figure 5:**
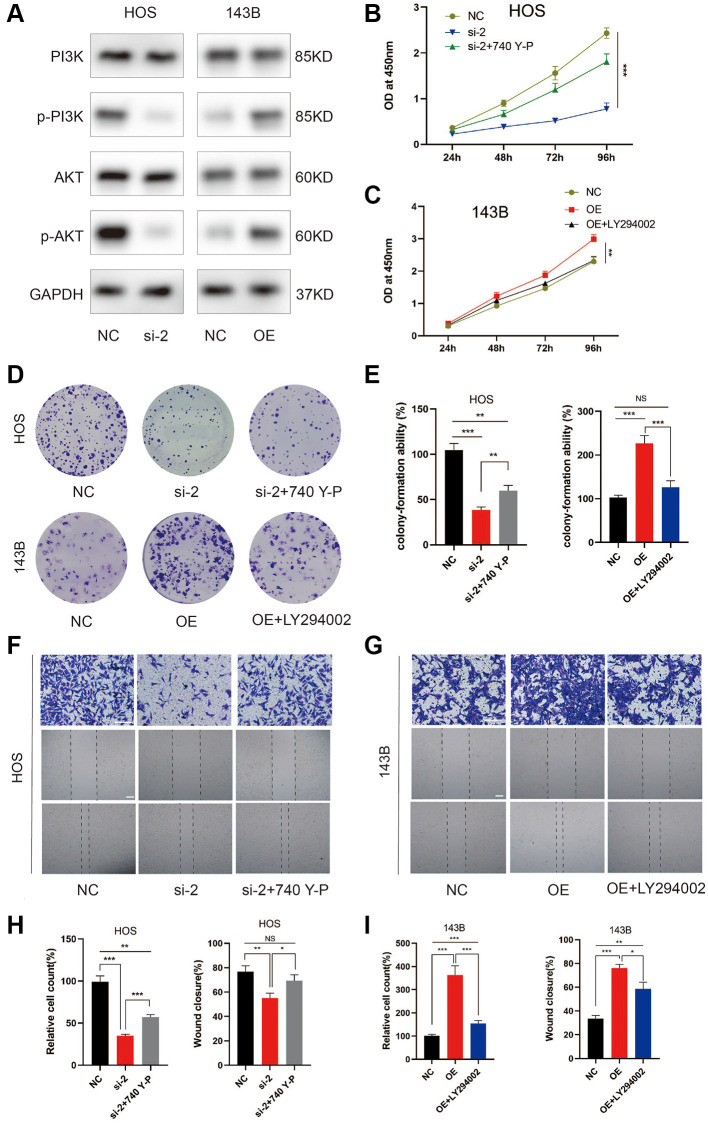
**ZCCHC12 contributed to OS progression through PI3K/AKT signaling pathway.** (**A**) The expression levels of PI3K/AKT signaling pathway-associated proteins were detected by western blot analysis. (**B**–**I**) functional rescue experiments using PI3K agonist 740Y-P or PI3K inhibitor LY294002 were performed to verify the effect of ZCCHC12 on proliferation and migration in OS. ZCCHC12-silenced HOS cells were treated with 740 Y-P while ZCCHC12-overexpressed 143B cells were treated with LY294002, then cells were used for proliferation analysis (**B** and **C**), CCK-assays; (**D** and **E**), colony formation assays) and migration analysis (**F**–**I**), Transwell migration assays and wound-healing assays). scale bar: 200 μm. *n* = 5; ^*^P < 0.05, ^**^P < 0.01, ^***^P < 0.001.

The KEGG analysis indicated the PI3K/AKT signaling pathway as the most enriched signaling pathway (Figure 1B). To assess whether PI3K/AKT pathway was involved in the effect of ZCCHC12 on OS, expression of PI3K, p-PI3K, AKT and p-AKT in OS cells were examined by western blot. The results showed that ZCCHC12 knockdown resulted in decreased PI3K and AKT phosphorylation, while ZCCHC12 overexpression strongly promoted their phosphorylation (Figure 5A). In addition, the rescue experiments confirmed that PI3K agonist 740Y-P attenuated ZCCHC12 knockdown’s inhibitory effects on OS cell proliferation and migration (Figure 5B, 5D–5F, and 5H), meanwhile, PI3K inhibitor LY294002 could reverse the promoting effect of ZCCHC12 overexpression on OS cell proliferation and migration (Figure 5C–5E, 5G and 5I). Taken together, these results suggested that ZCCHC12 contributed to OS progression by activating the PI3K/AKT signaling pathway.

### ZCCHC12 promoted OS growth *in vivo*

**Figure 6:**
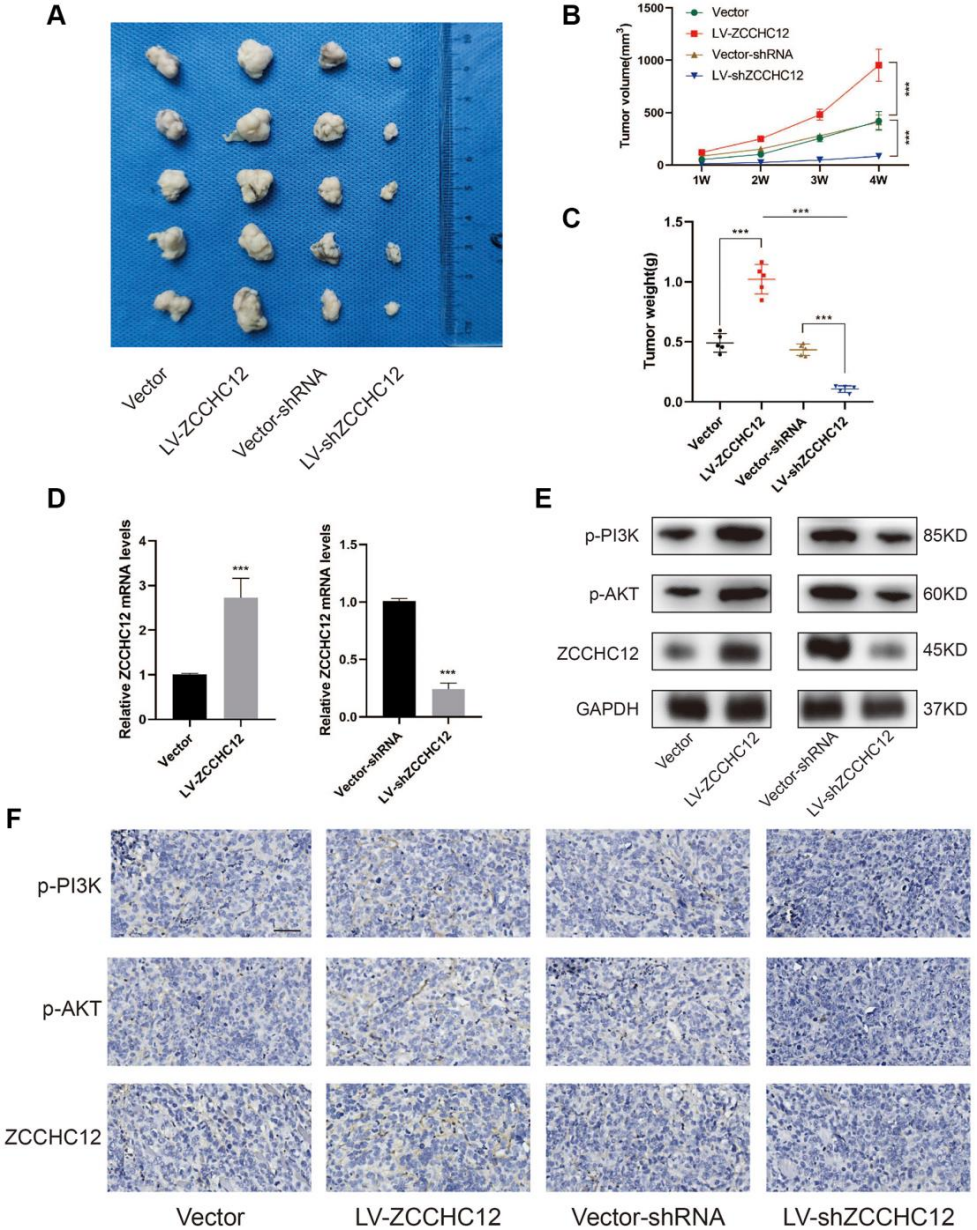
**ZCCHC12 promoted OS cells tumorigenicity *in vivo*.** (**A**) Subcutaneous tumors were removed from nude mice and fixed in 0.4% formalin. (**B** and **C**), Tumor volume and weights of xenografts in nude mice were measured. (**D**), qRT-PCR was applied to examine the expression of ZCCHC12 in tumor xenograft tissues. (**E**), Western blot was performed to examine the expression of p-PI3K, p-AKT and ZCCHC12 in tumor xenograft tissues. (**F**) IHC staining for p-PI3K, p-AKT and ZCCHC12 in tumor xenograft tissues. scale bar: 200 μm. *n* = 5; ^***^P < 0.001.

To investigate the *in vivo* role of ZCCHC12 in OS, we established nude mice xenograft model by subcutaneously injecting 143B cells stably transfected with LV-ZCCHC12, LV-shZCCHC12 or their empty vectors. Figure 6A–6C shows there was a significant increase in the volume and weight of the xenograft tumors after upregulation of ZCCHC12. In contrast, its downregulation showed opposite effects. The mRNA level of ZCCHC12 was detected by qRT-PCR (Figure 6D). The expression levels of p-PI3K, p-AKT and ZCCHC12 in xenograft tumors were measured by western blot and immunohistochemistry while p-PI3K and p-AKT are significantly inhibited in tumors tissues with ZCCHC12 knockdown as shown in Figure 6E and 6F.

## DISCUSSION

Osteosarcoma ranks among the most frequent primary tumors of bone. It has a high degree of malignancy, and remains a significant cause of cancer-related death among children and young adults [22]. At present, neoadjuvant chemotherapy followed by surgical resection and adjuvant chemotherapy is still the primary treatment approach for OS [23]. However, owing to the late of diagnosis, early metastasis and drug resistance of OS, its 5-year survival rate remains dismal [24, 25]. Therefore, a deep comprehension of OS molecular mechanisms may allow the identification of novel biomarkers, and is crucial for improving diagnosis and treatment.

The rapid advances in high-throughput sequencing has greatly improved our knowledge of the molecular mechanisms underlying carcinogenesis [26, 27]. In the present study, by analyzing a publicly available RNA-sequencing datasets from the GEO database, ZCCHC12 was found overexpressed in OS tissues than adjacent non-tumor samples. ZCCHC12 has been reported to serve as a transcriptional coactivator in the BMP pathway and was associated with some diseases such as thyroid carcinoma [16, 18–20]. There is, however, a lack of knowledge about the putative role of ZCCHC12 in OS.

Our present study revealed that ZCCHC12 played an active role in promoting OS progression. The expression of ZCCHC12 was much higher in OS cell lines compared to normal human osteoblasts (hFOB1.19). Functionally, ZCCHC12 could promote the *in vitro* proliferative and migratory abilities, as well as the *in vivo* tumorigenic capacity of OS cells. In addition, depletion of ZCCHC12 could induce OS cell apoptosis and S-phase arrest. Mechanistically, ZCCHC12 promoted the malignant progression of OS through activation of the PI3K/AKT signaling pathway.

The overall prognosis for patients with OS is poor because of its high metastatic potential [28]. EMT is a key mechanism of tumor metastasis, inhibition of which may attenuate cancer metastasis [29]. In this study, we found that silencing ZCCHC12 slowed down migration of OS cells. Furthermore, a positive correlation was found between the expression of ZCCHC12 and mesenchymal markers (N-cadherin, Snail and Vimentin), while a negative correlation was found between ZCCHC12 and epithelial marker (E-cadherin). The above results suggested that ZCCHC12 exerted a pivotal role in OS migration and EMT process. Thus, inhibiting ZCCHC12 may be an effective method for suppressing OS metastasis.

PI3K/AKT signaling has been shown to be frequently hyperactivated in OS, resulting in its initiation and progression [30]. We found that ZCCHC12 could enhance PI3K and AKT phosphorylation, while PI3K agonist 740Y-P and PI3K inhibitor LY294002 could rescue the effect of ZCCHC12 knockdown and overexpression, respectively. These findings revealed that PI3K/AKT signaling was activated by ZCCHC12 in OS. Therefore, combination therapy with the inhibition of PI3K/AKT pathway and ZCCHC12 through small molecule inhibitors may be a potential effective strategy for OS with high ZCCHC12 expression.

In conclusion, the present study identified ZCCHC12 as a novel player in OS for the first time, showing that ZCCHC12 could promote OS cell proliferative and migratory abilities through activating the PI3K/AKT pathway. Therefore, ZCCHC12 offers the prospect of being a novel molecular target for OS diagnosis and treatment.
